# Multifunctional neuron-specific enolase: its role in lung diseases

**DOI:** 10.1042/BSR20192732

**Published:** 2019-11-15

**Authors:** Cai-Ming Xu, Ya-Lan Luo, Shuai Li, Zhao-Xia Li, Liu Jiang, Gui-Xin Zhang, Lawrence Owusu, Hai-Long Chen

**Affiliations:** 1 Department of General Surgery, The First Affiliated Hospital, Dalian Medical University, Dalian 116011, Liaoning, China; Institute (College) of Intergrative Medicine, Dalian Medical University, Dalian 116044, Liaoning, China; 2 Department of Traditional Chinese Medicine, Dalian Obstetrics and Gynecology Hospital, Dalian 116021, Liaoning, China; 3 Department of Pediatric Orthopedics, Qingdao Women and Children’s Hospital, Qingdao 266035, Shandong, China; 4 Department of Biochemistry and Biotechnology, Kwame Nkrumah University of Science and Technology (KNUST), PMB, UPO, Kumasi, Ghana

**Keywords:** Chronic obstructive pulmonary disease, Enolase, Neuron-specific enolase, Pulmonary alveolar proteinosis, Solitary pulmonary nodules

## Abstract

Neuron-specific enolase (NSE), also known as gamma (γ) enolase or enolase-2 (Eno2), is a form of glycolytic enolase isozyme and is considered a multifunctional protein. NSE is mainly expressed in the cytoplasm of neurons and neuroendocrine cells, especially in those of the amine precursor uptake and decarboxylation (APUD) lineage such as pituitary, thyroid, pancreas, intestine and lung. In addition to its well-established glycolysis function in the cytoplasm, changes in cell localization and differential expression of NSE are also associated with several pathologies such as infection, inflammation, autoimmune diseases and cancer. This article mainly discusses the role and diagnostic potential of NSE in some lung diseases.

## Introduction

### Enolase

**Figure 1:**
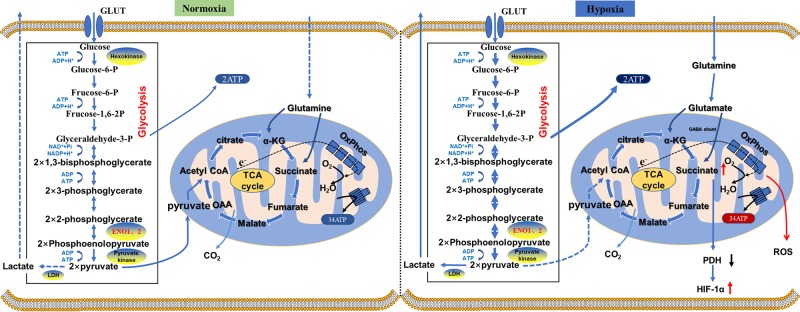
Enolase is a well-known glycolytic enzyme that catalyzes the interconversion of 2-phosphoglycerate and phosphoenolpyruvate during glycolysis and gluconeogenesis Enolase, in the glycolytic pathway, is involved in the breakdown of glucose into two pyruvate molecules and the high-energy compounds ATP and cofactor NADH, providing energy and material basis for cell metabolism and various life activities. Under normoxic conditions (left panel), most of the cellular energy is obtained from oxidative phosphorylation in the mitochondria with O_2_ as the terminal electron receiver in the electron transport chain to generate ∼34 ATPs/glucose molecule. However, in the absence of O_2_ or under reduced oxygen conditions (hypoxia, right panel), cells resort to substrate level phosphorylation by increasing the rate of glycolysis to generate ATPs from the process. The expressions and activities of glycolytic enzymes hexokinase, enolase and pyruvate kinase are elevated to facilitate the process. Cell damaging and cancer promoting factors, including ROS, are by-products of such inefficient cellular respiratory process.

Enolase (EC 4.2.1.11) is a well-known glycolytic enzyme that catalyzes the conversion of 2-phosphoglycerate into phosphoenolpyruvate and its reverse reaction during gluconeogenesis. Enolase is involved in the glycolytic pathway, which decomposes glucose into pyruvate and produces both high-energy compounds of ATP and cofactor NADH, providing energy and material basis for cell metabolism and various life activities (Figure 1). Therefore, enolases are among the most ubiquitously and abundantly expressed proteins in cells from archaebacteria to mammals, with highly conserved amino acid sequence [1,2]. Many studies have suggested that enolase, as well as pyruvate kinase and hexokinase, may dominate in metabolic contributions to inflammation and facilitate tumour proliferation under hypoxic conditions by elevating glycolysis [3,4].

**Figure 2:**
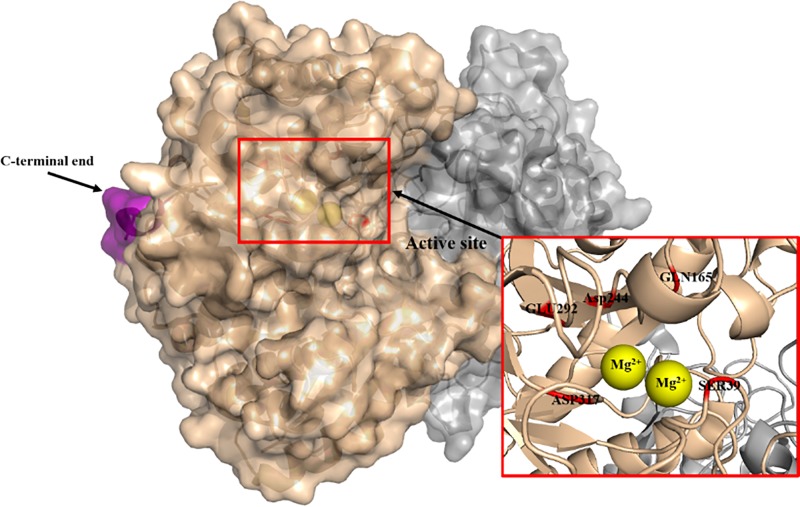
A simple analysis of space conformation of NSE: NSE is a metal-activated enzyme composed of two asymmetric γ subunits (brown and grey) Two types of Mg^2+^ binding sites contribute to catalysis. Mg^2+^ binding in site I (yellow above), traditionally called ‘conformational’, induces a conformational change in the enzyme and enables binding of substrate or substrate analogues. The second Mg^2+^ ion (yellow below), called ‘catalytic’, can bind in site II and then the catalytic reaction occurs. The second Mg^2+^ ion binds in the catalytic metal ion binding site in the closed conformation loop 36–43, which includes a residue crucial for catalysis: Ser^39^ (red). γ-enolase crystal structure (1TE6) was obtained from Protein Data Bank (PDB). The image was prepared by authors with Python.

Functionally, active enolases are dimers. They consist of non-covalently linked dimers of either α, β or γ subunits, which are expressed by different genes. These genes are not considered housekeeping genes because their expression is tissue-specific and varies during developmental, metabolic or pathophysiological conditions [5]. Besides the peptide molecules, enolases also require divalent metal ions to maintain their structural stability and catalytic activity, especially Mg^2+^ [6] (Figure 2). There are mainly three isozymes of enolase in vertebrate organisms: α-enolase (Eno1), β-enolase and γ-enolase, which are homodimers composed of two of the same subunits [1]. β and γ subunits readily form mixed dimers with the α subunit; the intermediate form is a hybrid molecule containing α and β/γ subunits. All possible dimers except βγ have been observed *in vivo*. The three enolase isoforms share high-sequence identity and kinetic properties [7–10]. Each subunit of enolase is made up of two domains: the smaller N-terminal extending from amino acid 1 to approximately 134 and the larger C-terminal domain (residues from approximately 143 to 434). The N-terminal domain has a β3α4 topology. The C-terminal domain has the eight-fold βα barrel structure with ββαα(βα)6 topology [10]. Between these domains is a short random structure fragment. This characteristic short variable region is situated predominantly on the surface of the molecule, far away from the active site. Additionally, residues that participate in catalytic activity are to a large extent conserved throughout [10,11]. The conservation of flanking residues on either side of these catalytic residues also indicates that the basic folding structure of all enolases is essentially the same [10]. α-Enolase exists in all fetal and most adult mammal tissues. However, it is replaced by other isoforms during tissue development [1]. The α-enolase levels in embryonic brain are high and decrease with the maturation of neurons. Conversely, γ-enolase levels are considered to be low in embryonic brain, and increase with the development of nervous system structure and function, indicating that there are two isoenzymes of enolase in mature brain: γ-enolase is proved to restrict to neuronal cells, hence the traditional name, neuron-specific enolase (NSE), while α-enolase is limited to glial cells (non-neuronal enolase, NNE) [12]. α-Enolase can be replaced by γ-enolase in the brain, which is a late event in the development of the nervous system and can be a marker of neural maturity [13,14]. Similarly, during ontogenesis, α-enolase is replaced by β-enolase in the muscle group [5]. The most significant difference between α-enolase (NNE) and γ-enolase (NSE) is the obvious lack of immunological cross-reactivity between the two proteins [15,16]. NNE is highly sensitive to chloride ions, urea and temperature. In contrast, NSE is significantly more stable against chloride-induced inactivation. NSE’s relative insensitivity to chloride ions is particularly interesting since chloride ions accumulate in nerve cells during repeated depolarization. The relative resistance of NSE to chloride ions may have evolved to adapt to the intracellular environment of neurons, thus preventing inactivation of chlorine-sensitive enolase in neurons when neurons depolarize, and interrupting glycolysis when metabolic energy is most needed [17]. Additionally, NSE has been proposed to exhibit higher or enhanced catalytic rate as such their preference by cancer cells and in obesity where there is increased energy demand, usually under reduced oxygen conditions [18,19]. Following the initial discovery and classification of γ-enolase as neuron specific, its presence in other non-neural cell/tissue types such as fibromuscular tissue of the prostate, lymphocytes, the myometrium of the uterus, spermatogonia, myoepithelial cells, the heart, and macula densa cells of the kidney, megakaryocytes and platelets has been empirically demonstrated [20].

Enolases are predominantly located in the cytoplasm of cells and exert catalytic effects. However, recent accumulation of evidence has revealed that they can also be detected in the nucleus, cell membrane and extracellular space, showing some other functions. For instance, α-enolase has been shown to act as a plasminogen receptor on the surface of several eukaryotic cells (such as neurons [21], monocytes [22], B cells, T cells and endothelial cells [23]), binding to plasminogen and protecting it from its inhibitor α-antiplasmin. The bound plasminogen is cleaved by specific protease to generate active plasmin, which degrades ECM, facilitating pathogen invasion, infiltration of inflammatory cells and migration of tumours [24] to participate in various pathophysiological processes including inflammation, myogenesis [25,26], tumorigenesis and angiogenesis [27]. α-Enolase also exhibited strong plasminogen binding activity on the surface of the prokaryote *Streptococcus pneumoniae* and contributes to its virulence potential during invasive infection [11]. Feo et al. [28] and Subramanian et al. [29] also observed α-enolase in the nucleus, playing an important role in the form of an alternative translation product as c-myc promoter-binding protein-1 (MBP-1). MBP-1 binds c-myc P2 promoter and negatively regulates transcription of the protooncogene. Furthermore, α-enolase has also been described as a heat-shock protein [30] and a hypoxic stress protein [31].

β-Enolases may segregate to different subcellular sites where they can respond to specific functional demands. In muscle cells, the structural features of the β-enolase allow it to localize in the naked areas of actin thin filaments (the I band of the sarcomere) to furnish the ATP for muscle contraction along with other glycolytic enzymes [32].

### NSE

γ-Enolase, also known as NSE, is a soluble cerebral protein, first described by Moore and McGregor in 1965 [33]. It is mainly located in the cytoplasm of central and peripheral neurons and neuroendocrine cells [34]. Small amounts of NSE were also found in non-neuronal and non-neuroendocrine cells or tissues, such as platelets, erythrocytes, prostate, breast tissue and uterus [20,35]. The organ localization of NSE results in the elevation of its serotype associated with various nerve damages such as ischaemic stroke, intracerebral haemorrhage, post-traumatic brain injury and spinal cord injury. Thus, the level of NSE measured in blood and cerebrospinal fluid may be a potentially useful marker for assessing neuronal death in different centre neuronal system injuries, with increased correlation to injury severity [36]. Expression of cell surface γ-enolase initiates inflammatory reaction after injury. Following injury, NSE rapidly transfer from the cytosol of cells (e.g., neurons, glial cells and inflammatory cells) to the surface. On the one hand, it enhances cell antigen presentation and degrades ECM through concentrating plasminogen on the surface; on the other hand, NSE could drive the production of ROS, NO and various cytokines (such as TNF-α, IL-1β, INF-γ, TGF-β and MCP-1) via pro-inflammatory signaling pathway to bolster inflammation [27,37–39]. Additionally, NSE can play a protective role in promoting cell survival and act as neurotrophin in neurons. Its hydrophobic domain in the N-terminal region enables it to dock on the surface of plasma membrane of neurons, glia and astrocytic cells. The C-terminal part of the molecule could promote neuronal survival, differentiation and axonal regeneration by activating the signalling pathways of phosphatidylinositol-4,5-bisphosphate 3-kinase (PI3K) and mitogen-activated protein kinase (MAPK). NSE-mediated PI3K activation also regulates RhoA kinase [40], which can influence actin cytoskeleton reorganization, induction of neurite outgrowth, and growth arrest in neuronal cells, for negative regulation [27,37]. This additional function could be important also in cancer cells (as discussed later). Several studies have confirmed the ability of a synthetic peptide of 30 amino acids mimicking the C-terminal part of NSE to promote survival of rat neocortical cells *in vitro* [41]. While cathepsin X can abolish this neurotrophic activity by cleaving the C-terminal of NSE, γ-1- syntrophin enhances this neurotrophic activity by trafficking NSE towards the plasma membrane [42,43]. In addition, Hafner et al. [44] observed large amount of C-terminally cleaved form of γ-enolase around the lesion (amyloid plaque) in the brain of transgenic mice of Alzheimer’s disease while its intact form mainly existed in microglia cells near to senile plaque. It was confirmed in mouse microglial cell line that amyloid-β peptide can induce up-regulation of γ-enolase, which plays a protective role in amyloid-β-related neurotoxicity. Furthermore, Pislar et al. [45] also found that the C-terminal of NSE can impair amyloid-β-induced apoptosis by interacting with p57 neurotrophin receptor (p57^NTR^) to inhibit the activation of its downstream signalling factors. These experimental results showed that although γ-enolase on the cell surface is not like α-enolase which have C-terminal lysine binding to plasminogen [46], its C-terminal specific amino acid sequence can play dual role in the pathophysiological process of the nervous system through multiple signal pathways [37,42,43,47]. In addition to membrane localization of γ-enolase, studies from Soh have also observed a significant nuclear localization of NSE in Cd^+2^- and As^+3^- transformed breast epithelial and urothelial cell lines. When NSE expression in a given cell is low, it tends to be localized in the nucleus, while when NSE expression is high, it is localized in both the nucleus and cytoplasm. The discovery of the nuclear localization indicates that enolase functions extend far beyond glycolytic activity [35,48].

γ-Enolase might also have a multifunctional role in cancer progression. As a cytoplasmic enzyme, it provides energy support for the survival and unlimited proliferation of cancer cells through increased aerobic glycolysis (Warburg effect, Figure 1) [49–51]. Moreover, Vizin and Kos [52] have revealed a new mechanism of γ-enolase involvement in cancer progression: upon stimulation, the cellular localization of γ-enolase changes, and can traffic to the cell surface to activate survival promoting signaling pathways as it does in neuronal cells and to promote migration of tumour cells. This in effect helps tumour cells adapt to stressful conditions (hypoxia, chemotherapy, radiotherapy and serum starvation). For instance, existing experiments have found that when glioblastoma cells were in a state of serum starvation and hypoxia, the expression of γ-enolase in the cells was significantly increased, and the expression level of survival promoting proteins as well as anti-apoptosis signal pathways (which are triggered by γ-enolase as in neurons) were significantly elevated [53–55]. In addition, Yan et al. [53] observed that knocking-out γ-enolase significantly reduced migration of tumour cells. It may be related to the function of γ-enolase in promoting dynamic remodelling of actin cytoskeleton, which is an important prerequisite for cell migration [56]. The precise mechanism by which NSE induce actin remodelling is currently not very clear. The author regarded that γ-enolase may induce actin polymerization and redistribution by interacting with actin filaments via γ-1-syntrophin and regulating RhoA kinase (A regulator of actin cytoskeleton organization) [37,43,57]. γ-Enolase is usually overexpressed in tumours of neurogenic and neuroendocrine origin and is used as a marker for detecting neuroendocrine differentiation of tumour cells. At present, many studies have shown that γ-enolase is of certain significance in the diagnosis, treatment monitoring and prognosis of various neuroendocrine tumours, especially lung cancer.

## NSE assays in tissues and body fluids

Assays for NSE concentration and activity determinations have seen continuous innovation with the progress of science and technology. Several qualitative or quantitative techniques have been developed to detect NSE in tissues and body fluids (serum, cerebrospinal fluid, urine etc.).

### Radioimmunoassay

As early as the 1970s, a solid-phase radioimmunoassay (RIA) using ^3^H-labelled antigen and double antibody to determine NSE and NNE in brain tissue was described by Marangos et al. [13,58,59]. It was also widely used for the determination of NSE in other tissues, but it was not sensitive enough to measure NSE of nanogram amount in body fluids such as cerebrospinal fluid and cell culture fluid [58,59]. Parma et al. [60] proposed an improvement by labelling antigen with [125] I. Because the activity of NSE labelled with [125] I was higher than that with ^3^H, the improved sensitivity allowed the determination of low amounts of proteins in body fluids. In 1989, Paus and Nustad [61] applied monoclonal antibody against NSE and mono-disperse magnetizable particles (as solid phase carriers) to immunoradiometric assay (IRMA). The measurement range of this test was 0.4–170 μg/l. Compared with RIA using polyclonal antibodies, the incubation time was shorter, with higher sensitivity and accuracy [62]. RIA is a traditional method for detecting tumour markers. It has the advantages of high sensitivity, easy commercialization and so on. However, compared with other methods, it has the disadvantages of short service life of kits, radioactive pollution risks and so on. At present, it has gradually been replaced by other detection methods.

### Enzyme immunoassay

In 1987, Anastasiades et al. [63] developed an enzyme-linked immunosorbent assay (ELISA) using double antibody sandwich method [64] to measure NSE concentration in serum of patients with small cell lung cancer (SCLC). The sandwich used in this system was rabbit anti-rat enolase that cross-reacts with the human γ monomer, resulting in the test being specific for only the γγ isoenzyme. An avidin–biotin–peroxidase complex system was used to provide increased assay sensitivity. Following Schmitt’s two-step enzyme immunoassay (EIA) using a specific monoclonal antibody against NSE in conjunction with a polyclonal antibody to measure NSE level in serum [65], Ebert et al. proposed a new detection method at the beginning of the 20th century: the New Elecsys NSE assay, which is a single-step, solid-phase EIA of a sandwich type employing two specific monoclonal antibodies raised in mice against purified γ-enolase from human brain [66]. They assessed its utility as a sensitive and specific test for the diagnosis of SCLC and hold the view that the Elecsys NSE ELA is a reliable and accurate diagnostic procedure to measure NSE of serum samples with a wide measuring range (up to 370 ng/ml) and a short incubation time (18 min).

### Immuno-bioluminescent assay

In 1983, Wevers et al. [67] determined NSE in human serum and cerebrospinal fluid by bioluminescent assay using luciferine–luciferase system. A solid-phase immune-bioluminescence assay for NSE in human plasma was also developed by Gerbitz et al. [68] in 1984. Purified antibodies against NSE and NNE are coated on to polystyrene tubes. Each sample containing the isoenzyme activity to be determined is incubated in the respective coated tube. The coating is then washed and the reaction of the antibody-bound enzyme is initiated by adding 2-phosphoglycerate, ADP and pyruvate kinase. ATP formed in the enzymatic reaction is measured by following the increase in light emission in the firefly luciferase bioluminiscent system. In 1986, Viallard and colleagues [69,70] proposed two new methods using bioluminescence assay for the determination of NSE in serum. The key steps for these two assays were immunocapture of enolase isoenzymes containing γ subunit and electrophoretic separation of the isoenzymes on cellulose acetate respectively. At present, adenosine triphosphate bioluminescent assay is widely used in microbial biometrics. Its role in tumour marker detection is gradually replaced by chemiluminescent immunoassay.

### Chemiluminescence analysis

Chemiluminescence immunoassay uses chemiluminescent substances (e.g., luminol) or enzymes (e.g., alkaline phosphatase, ALP) as labels to directly label antigens or antibodies, forming immune complexes through antigen–antibody reaction. An oxidant or enzyme luminescent substrate is added to form an intermediate in an excited state which could emit photons to return to a stable ground state. The intensity of luminescent signals is proportional to the amount of substances being measured. Fu et al. [71] developed a chemiluminescence EIA based on magnetic nanoparticles (MPs-CLEIA) in 2012 to detect NSE in SCLC patients’ serum. The method connected fluorescein isothiocyanate (FITC) labelled NSE capture antibody and NSE with ALP labelled detection antibody in a sandwich-type manner. This immune complex reacted with anti-FITC coated magnetic beads. Due to the enrichment effect of magnetic field, the method showed high sensitivity (LOD < 0.2 ng/ml) and excellent accuracy, stability (CVs < 10%), and specificity. Compared with the traditional CLEIA method, MPs-CLEIA method was superior. As a result, the MPs-CLEIA method is expected to become a rapid, sensitive and reliable diagnostic for SCLC in large-scale screening due to its advantages of low cost and easy automation.

### Immunosensor

Natural enzymes that label antigens/antibodies have the disadvantages of poor stability, limited sources, sensitivity to environmental changes, susceptibility to environmental influences and denaturation, and the labelling process usually damages the biological activity of antibody molecules. Therefore, enzyme-free immune systems based on metals and metal complexes, magnetic nanoparticles and quantum dots are continuously being developed. Immunosensors have always been concerned and favoured by tumour researchers. The biosensor combines specific immune reaction with biosensing technology, and the biological recognition part of the biosensor comes from the specific recognition and binding effect of antigen and antibody. Biological signals are converted into electrical signals for detection through a physicochemical transducer and a signal amplification device. Metal nanomaterials are often used to construct immunosensors due to their unique optical, electronic and catalytic properties. Ho et al. [72] developed an electrochemical sandwich immunosensor. This Au nanoparticle congregate-based assay provides an amplification approach for detecting Eno1 at trace levels, leading to a detection limit as low as 11.9 fg. In addition, carbon nanomaterials and polymer composites are also widely used in the manufacture of immunosensors due to their good mechanical properties and redox properties. In 2013, Li and Tian [73] developed a new and enzyme-free electrochemical immunoassay protocol for the sensitive electronic monitoring of NSE on a monoclonal mouse anti-human NSE antibody (mAb)-modified glassy carbon electrode, using guanine-decorated graphene nanostructures (GGNs) as nanotags. Recently, Wei et al. [74] used an Au nanoparticle/reduced graphene oxide composite (AuNP-RGO), a signal-enhanced electrochemical immunosensor without label to detect NSE. In addition to the above materials, other materials with different functions are often introduced to improve the performance of immunosensors. For instance, quantum dots [75] are used to improve sensor sensitivity for their high surface activity, small size, and sensitivity to light, electricity and temperature. Sensors constructed with mesoporous material with good pore structure and interface structure [76] can maintain good activity and functionality of the enzyme. Sensors consisting of hydrogel [77] have good stability and high solubility, and can respond to external stimuli to conduct corresponding changes.

### Proteomics

Recently, Zhong et al. [78] developed an on-chip spyhole nanoelectrospray ionization mass spectrometry for sensitive biomarker detection in small volumes. This electrospray ionization mass spectrometry uses electrospray to liquidize polypeptides for identifying proteins. This method can ensure the integrity of the sample molecules during ionization and will not fragment ions. Protein chip is a newly developed analysis technology in the past decade. Protein probes are arranged on the surface of supports to capture target proteins, and then qualitative or quantitative analysis is carried out by detectors. Clinically, it is often used to screen and find tumour markers. Liu et al. [79] designed a bead-based microarray chip using a single layer of polydimethylsiloxane (PDMS) as carriers. The assay could be used for simultaneously detecting three lung cancer biomarkers: carcinoembryonic antigen (CEA), fragments of cytokeratin 19 (CYFRA21-1) and NSE in 10 μl of human serum, with a wide linear dynamic range (1.03–111 ng/ml for CEA and CYFRA21-1; 9.26–1000 ng/ml for NSE) and a low detection limit (CEA: 0.19 ng/ml; CYFRA21-1: 0.97 ng/ml; NSE: 0.37 ng/ml; S/N = 3). Furthermore, the current approach is easy to operate without extra driving equipment such as pumps, can make parallel detection for multiplexing with rapid binding kinetics, small reagent consumption and low cost.

## γ**-Enolase in lung cancer**

γ-Enolase is expressed only in specific tissues under physiological conditions. Overexpression of NSE and increased level in serum may be related to the malignant proliferation of these tissues, and therefore could be of value in diagnosis, staging, treatment and prognosis of such cancers, especially lung cancer.

According to a systematic analysis from the Global Burden of Disease Study, the number of cases of trachea, bronchus and lung cancer increased by 28% between 2006 and 2016, 2 million people suffering in 2016, and resulting in 1.7 million deaths, making it the most common and lethal human cancer in the world [80]. Early stage diagnosis of lung cancer is of great significance in clinical work. Primary bronchogenic carcinoma (lung cancer) is generally classified into four major cell types by histology: squamous cell lung cancer (SQC), lung adenocarcinoma, large cell lung cancer and SCLC. The first three types are grouped together as non-SCLC (NSCLC) [81]. The differences in behaviour, pathological and clinical characteristics between SCLC and NSCLC contribute to their diagnosis, treatment and prognosis. In addition to histology, an alternative diagnostic method also may be critical, especially if it is based on simple laboratory tests, performed on serum or other body fluid samples.

### SCLC

SCLC is a subtype of lung cancer with extremely high malignancy, accounting for approximately 15–20% of lung cancer [82]. It often occurs in the middle and old age, with more men than women, and is characterized by its early metastasis, rapid growth and sensitivity to initial chemotherapy [82]. Besides the TNM staging of tumour [83], the ‘two-stage’ method is usually used in clinical research and practice of SCLC. In 1973, VALG proposed two clinical stage of SCLC: limited-stage disease (LD) and extensive-stage disease (ED) [40]. In LD, the lesion is limited to one side of thoracic cavity and can be included in a radiotherapy field. In ED, the tumour has metastasized to the contralateral chest and distant sites. Most of the cases diagnosed are ED at first visit, while LD cases are relatively few (15–25%). SCLC is an aggressive neuroendocrine tumour; therefore, neuroendocrine markers such as NSE, chromogranin A (CGA) and pro-gastrin-releasing peptide (ProGRP) have been shown to be important to immunohistochemically characterize these malignant lung cancer and can be used as tumour markers released into the circulation [84].

#### Diagnosis

NSE is currently recognized as the most reliable tumour marker in the diagnosis of SCLC. Its serum level differed significantly according to tumour size, disease stage and distant metastasis (all *P*<0.05), while no association was found with gender or age (both *P*>0.05) [85]. Kostovski et al. [86] performed a basic immunohistochemical study of four antigenic phenotypes (TTF1, CK7, CK20 and NSE) in 21 patients with lung carcinomas and observed that NSE(+) was found in all cases of SCLC (100%). Another group of experiments reported similar results: the expression of γ-enolase in tumour tissues of SCLC patients was significantly increased, 35-times higher than that of normal tissues, and the γ/α+γ value in lung tissues of SCLC patients was significantly higher than that of normal tissues (*P*<0.01) [87]. In addition, NSE levels in serum are also significantly different between patients and non-patients: increased level of serum NSE (>13 ng/ml) was detected in 68% of patients with limited SCLC and 87% of patients with extensive SCLC [88]. Huang et al. [89] conducted a meta-analysis of serum NSE levels to establish an evidence-based perspective on its clinical value for screening patients with SCLC. Pooled sensitivity of NSE for detecting SCLC was 0.688 (95% CI: 0.627–0.743), specificity was 0.921 (95% CI: 0.890–0.944), positive likelihood ratio was 8.744 (95% CI: 6.308–12.121), negative likelihood ratio was 0.339 (95% CI: 0.283–0.405). The diagnostic performance was better in Europe than in Asia [sensitivity: 0.740 (95% CI: 0.676–0.795) vs. 0.590 (95% CI: 0.496–0.678), specificity: 0.932 (95% CI: 0.904–0.953) vs. 0.901 (95% CI: 0.819–0.948)]. And ELISAs had the highest sensitivity and RIA had the highest specificity. Therefore, serum NSE has high diagnostic efficiency in the early detection of SCLC, but its curative effect varies according to different research region and assay methods. Some studies suggested that tumour markers in exhaled breath condensate (EBC) may have better diagnostic performance for lung cancer than those in serum. The cut-off values of NSE in serum and EBC determined in the present study were 14.30 and 4.65 ng/ml, respectively. The overall correct prediction percentage was 57.76% for serum-NSE and 59.01% for EBC-NSE, and NSE had the highest predictive positive rate in SCLC (62.50% in serum; 87.50% in EBC) [90]. Even though numerous studies have confirmed the diagnostic value of NSE in SCLC, it is not an ideal biomarker; its sensitivity is relatively low. Some authors regard that the combined diagnosis of marker combinations can effectively improve the accuracy of diagnosis. In SCLC patients, diagnostic utility of other tumour markers, such as haptoglobin, CEA and CYFRA 21-1 was also confirmed [91]. Wang et al. [92] proposed that the combined detection of Hp, CEA, NSE and CYFRA21-1 could significantly improve the sensitivity and specificity of lung cancer diagnosis and could be used for pathological typing. The combination of four tumour markers could produce a positive detection rate of 85.0%, which is significantly higher than that of any single test. An area under the ROC curve (AUC) analysis showed that the positive detection rate of Hp and CYFRA21-1 was higher than other markers for squamous cell carcinoma. In adenocarcinoma cases, the positive detection rate of CEA is higher than other markers. For small cell carcinoma, NSE has the highest positive detection rate [92]. Jiang et al. [93] also found that the detection of TK1 combined with CYFRA21-1, CEA and NSE increased the diagnostic value of TK1 for lung squamous cell carcinoma, adenocarcinoma and SCLC, respectively.

#### Monitoring treatment

Increasing researches are focussed on the usefulness of various tumour markers in evaluating SCLC chemotherapy response. Liu et al. [94] investigated the associations of serum levels of lactate dehydrogenase (LDH), ProGRP and NSE with clinical response and survival in SCLC patients receiving first-line platinum-based chemotherapy. Of the 136 SCLC patients who received first-line platinum chemotherapy, 97 patients achieved complete relief (CR) and partial relief (PR), with an overall response rate of 71.3%. Compared with patients in stable disease (SD) and progress disease (PD), NSE and LDH level declined in patients who achieved CR + PR. Multivariate regression analysis revealed that NSE > 50.324 ng/ml and distant metastases were independent risk factors for patients achieving CR + PR and were independently correlated with worse overall survival (OS). The authors concluded that there is a promising role for NSE and LDH in predicting therapy response and survival of SCLC patients receiving first-line platinum-based chemotherapy. A lot of studies have observed that NSE levels are rapidly normalized in SCLC patients receiving combined chemo- and radiotherapy [85,95–97]. The reasons for the phenomenon are difficult to explain. Perhaps, it should be associated with the influence of chemotherapeutics on enolase activity and disorders of anaerobic glycolysis. Núria et al. have developed a model for biomarker that, without using tumour size data, is capable of predicting disease progression assessed by CT scans (RECIST data) in SCLC patients [98]. They successfully applied this framework to data regarding LDH and NSE concentrations in patients diagnosed with SCLC and believe that the proposed modelling framework of circulating biomarkers could constitute a powerful additional strategy for disease monitoring in SCLC patients.

#### Prognosis

Even though SCLC is initially sensitive to radiotherapy and chemotherapy, secondary multi-drug resistance is extremely easy to occur. The majority of patients have poor prognosis and eventually die of tumour recurrence. Data from the Moffitt Cancer Center showed that median survival (MS) time of patients with SCLC is poor, approximately 25.1 months in LD and 10.4 months in ED patients. Five-year survival rates are 9.9% and lower than 3% in LD and ED patients, respectively [99]. Petrovic et al. [84] analyzed the effects of circulating neuroendocrine markers CGA, ProGRP and NSE, in addition to other more classical prognostic variables, on survival duration of SCLC patients. MS of the entire study population (97 untreated patients from a single-centre and histologically proven SCLC, of which 51.5% were LD patients) was 13 months. Univariate Cox regression analysis found that survival rate significantly correlated with performance status (PS), disease stage, CGA, ProGRP and NSE levels, while age and sex did not affect prognosis. An ECOG performance status ≥ 2, extensive stage disease, a serum CGA level > 56 ng/ml, a serum ProGRP level > 58 pg/ml, and a serum NSE level > 19 ng/ml would lead to a shorter survival time of patients. The study carried out in 2016 by Huang et al. [85] supported the previous theory and emphasized NSE in serum as a better prognostic factor than Pro-GRP, because it is a prognostic factor independent of disease stage. According to some authors, an increase recurring in NSE level in serum of SCLC patients after chemotherapy may be related to the relapse of the disease. At relapse, 20–60% of patients had elevated serum levels of NSE [100–102], and the rate of CR to salvage chemotherapy in those patients was significantly lower than patients without elevated levels of NSE (2.2 vs 26.7%; *P*=0.001) [102]. These results show that serial NSE measurements are useful for the early prediction of SCLC relapse and for early administration of salvage chemotherapy for affected patients.

A series of studies document the value of NSE in the diagnosis, monitoring and evaluation of treatment response in SCLC patients, as well as the prognostic and predictive values of this biomarker. A summary of the information obtained both from literature and from our studies on the utility of NSE in diagnosing SCLC patients is contained in Table 1.

**Table 1:** Basic information about the utility of NSE determinations in SCLC

Utility of NSE	References
**Diagnosis**	
NSE: helpful in the assessment of lung cancer, histological type, differential diagnostics; the marker is mostly used in SCLC diagnosis	[86,89,96,87]
In SCLC patients, serum NSE levels are significantly different due to tumour size, disease stage and distant metastasis	[85]
**Predictive value**	
NSE is a sensitive marker for therapeutic monitoring of lung cancer	[96]
Serial NSE measurements are advantageous for early prediction of relapse in SCLC patients	[101]
At relapse, the serum level of NSE is a useful predictive marker for CR to salvage chemotherapy	[102]
**Prognostic value**	
In SCLC patients, NSE is a better prognostic factor in comparison with ProGRP	[85]
For SCLC patients who did not receive treatment - the serum NSE level > 19.0 ng/ml shorter MS	[103]
For SCLC patients receiving first-line platinum-based chemotherapy, NSE is of great predictive value for the therapy response	[94]
Before treatment - high NSE serial concentration (>50.324 ng/ml) – unfavourable prognostic factor	
For patients with SCLC who have achieved a CR or PR to first-line chemotherapy, the serum level of NSE is a useful prognostic factor after relapse	[102]

### NSCLC

Although NSCLC is a common clinical pathological type of lung cancer, accounting for approximately 80–85%, its malignancy is relatively low. For early NSCLC cases, the 5-year survival rate can be significantly improved by standard surgical treatment [104]. However, it is difficult to make early diagnosis for NSCLC. Most patients are at advanced disease at diagnosis, thus missing the best time for radical surgery. Therefore, it is very important to establish a complete screening system for NSCLC for its early diagnosis and treatment [82,105,106]. As mentioned earlier, NSE is the first choice in tumour markers for the diagnosis of SCLC, but there are still partial NSCLC patients with elevated serum NSE levels clinically [107]. Dong et al. [108] found that CEA and NSE in serum are potentially effective biomarkers for diagnosing NSCLC. The diagnostic sensitivity and specificity were 66.67 and 78.69%, respectively, for serum NSE at the cut-off value of 19.35 ng/ml; the diagnostic area under the ROC curve was 0.76 for NSE. However, some studies have suggested that in patients with peripheral pulmonary carcinoma, the detection of tumour markers in BALF had more diagnostic value than serum samples [109]. In addition, some studies found that in patients with advanced NSCLC who have lost the opportunity for surgery and were treated with gefitinib (an epidermal growth factor tyrosine kinase inhibitor, EGFR-TKI), the progression-free survival (PFS) and OS significantly reduced with increased in plasma NSE level before treatment, indicating that high pre-treatment serum level of NSE predicted poor EGFR-TKI therapeutic effect. Thus, it could be clinically useful in patients with NSCLC scheduled to receive gefitinib treatment [110,111]. At present, the value of NSE in judging prognosis of NSCLC is still controversial. Some believe that pre-treatment serum NSE level is an important prognostic factor of advanced NSCLC. In a recent study involving 224 patients, Wang et al. [112] evaluated the prognostic significance of serum tumour markers (CYFRA 21-1, CEA and NSE) in locally advanced squamous cell carcinoma of lung (LA-SCCL) after radiotherapy and found that increased NSE predicted poor distant metastasis-free survival (DMFS). Another analysis by Chen et al. [113] and Nisman et al.’s [103] team confirmed this conclusion, and found that a higher level of NSE before treatment was closely related to brain metastasis of advanced NSCLC. The serum level of NSE in 28 patients with brain metastasis was significantly higher than that of 98 patients without metastasis (34.18 ± 28.48 vs. 13.87 ± 4.49 ng/ml, *P*<0.05). Zhou et al. [114] also found that age, pathological type and serum NSE concentration of lung cancer patients are independent risk factors for bone metastasis. The NSE serum concentration of patients with bone lesions is significantly higher than those without bone metastasis (39.18 ± 62.18 vs. 29.16 ± 40.21 ng/ml, *P*=0.018) [114]. However, other studies have proposed that serum NSE level in NSCLC patients has no prognostic significance [115,116].

## NSE and tuberculosis

As early as 2009, Racil et al. [117] proposed the diagnostic value of NSE in pulmonary tuberculosis (TB). They conducted a prospective study and collected serum levels of four tumour markers including NSE, cancer antigen 125 (CA125), ACE and CYFRA21-1 before anti-TB chemotherapy in 40 male TB patients (during 2005–2007). They found the levels of NSE were high in 91.66% of cases with an average value of 29.22 ng/μl (2.24 × normal). This highest sensitivity was superior to those of other tumour markers: 55.55% for CA125, 28.94% for ACE and 7.6% for CYFRA21-1. They thought the highest sensitivity of the NSE in pulmonary TB, with no neoplastic pathology could be interesting for diagnosis of smear negative TB, with small amounts of Bacilli. In addition, several studies have found that the levels of NSE, S100B and Neuropeptide Y (NPY) in serum and cerebrospinal fluid of children with acute miliary TB secondary to tuberculous meningitis are significantly higher than those of children with acute miliary TB or meningitis alone [118,119]. The early detection of these tumour markers is of great significance for the diagnosis of tuberculous meningitis secondary to acute miliary TB.

Nam et al. [120] discussed the predictive value of NSE in indicating TB activity and severity and determined the origin of NSE in TB patients. They conducted a single-centre retrospective analysis of newly diagnosed TB patients from the years 2010 to 2011. According to chest X-ray, patients were divided into two disease groups (focal segmental or extensive). Pre- and post-treatment NSE concentrations were evaluated. In order to determine the origin of serum NSE, NSE staining was compared with macrophage-specific CD68 staining in lung tissue and tissue microarray using immunohistochemistry and immunofluorescence. The results showed that the serum concentration of NSE increased significantly in TB patients, and NSE level decreased after treatment (*P*<0.001). The average serum concentration of NSE in the extensive group (25.12 ng/ml) was significantly higher than that in the focal segment group (20.23 ng/ml, *P*=0.04). Immunohistochemical staining showed that a large number of macrophages were positive for NSE and CD68 in TB tissues. In addition, NSE signals mainly co-located with CD68 signals in tissue microarrays of TB patients. The authors regarded that NSE could be used to monitor TB activity and therapeutic response and that elevated serum NSE level is, at least in part, derived from macrophages in granulomatous lesions.

## NSE and chronic obstructive pulmonary disease

Barouchos et al. [121] investigated the correlation between tumour markers and inflammatory biomarkers in patients with chronic obstructive pulmonary disease (COPD) exacerbation. Referring to the Global Initiative for Chronic Obstructive Lung Disease (GOLD), patients were categorized into one of two disease groups (severity C and D). As a result, in group C, there was a significant positive correlation between C-reactive protein (CRP) and CA125 (*P*=0.05) while in group D, there was a significant positive correlation between white blood cells count (WBC) and NSE (*P*=0.02), between CRP and Cancer antigen 19-9 (CA19-9) (*P*=0.02) and NSE (*P*<0.001), and between the erythrocyte sedimentation rate (ESR) and NSE (*P*=0.03). In contrast, there was no significant difference in the two groups for NSE, CEA and CYFRA21-1. These results suggested that certain tumour markers were increased and were associated with increased levels of inflammatory biomarkers and with disease severity.

## NSE and solitary pulmonary nodules

With the development of imaging technology and low-dose spiral CT chest screening technology, the number of solitary pulmonary nodules (SPNs) found in clinics is gradually increasing. The benign lesions of SPN are mostly infectious granuloma, followed by TB and hamartoma. The malignant lesions are mainly lung adenocarcinoma and squamous cell carcinoma. It is of great significance to identify the benign and malignant SPN as early as possible. Chu et al. [122] investigated the potential diagnostic values of CEA, SCC, CYFRA21-1 and NSE for lung cancer in patients with suspicious pulmonary masses in an extensive and large-scaled population. A combination assay of SCC, CEA, CYFRA21-1 and NSE increased sensitivity to 43.4%, which was still low in diagnosing lung cancer but was higher than any other single tumour marker. However, these results are inconsistent with that of Ni and Liu [123]. In their study, the difference in NSE positive rate and serum level between benign and malignant SPN had no statistical significance. The area under ROC curve was less than 0.5, which is not of diagnostic value for SPN. This difference could be due to the small proportion of SCLC in their cases.

## NSE and pulmonary alveolar proteinosis

Pulmonary alveolar proteinosis (PAP) is an extremely rare interstitial lung disease characterized by the abnormal alveolae accumulation of a large number of lipoproteinaceous substances leading to impairment of lung ventilation function and increased risk of respiratory infections. The aetiology is still not clear and may be related to functional defects in alveolar macrophages. The diagnosis of PAP is easily missed or confused with other interstitial lung diseases with similar manifestations since it lacks typical clinical symptoms. Currently, open-lung biopsy and bronchopulmonary biopsy are used as the gold standard for clinical diagnosis but it is less required because BALF and imaging examination also have great significance for diagnosis. In 2016, Mo et al. [124] analyzed the clinical, pathological and biochemical characteristics of 11 patients with PAP in order to provide more information on diagnosis and management of PAP. They observed that CEA increased in most patients and CYFRA21-1 and NSE increased in all patients. Similar results were also obtained in a study by Fang et al [125]. in serum of patients with PAP, the changes of tumour markers (CEA, SCC and NSE) were consistent with the changes of severity index (LDH and PaO_2_). Especially, significant positive correlations were found between levels of CEA and NSE in serum and LDH values (r = 0.60, *P*<0.001 and r = 0.56, *P*<0.001, respectively). After whole lung lavage (WLL), the levels of CEA, NSE and SCC in serum decreased significantly (15.7 ± 22 vs. 8.7 ± 10.6, 16.6 ± 11.8 vs. 7.9 ± 5.2, 0.59 ± 0.42 vs. 0.4 ± 0.24; *P*<0.05 respectively). In addition, Arai et al. [126] also specifically analyzed the correlation between CYFRA21-1 and other disease severity markers of PAP, including pulmonary function parameters, alveolar–arterial oxygen gradient, British Medical Research Council score reflecting shortness of breath and disease severity score. They observed that serum CYFRA 21-1 level at diagnosis was significantly correlated with the measured disease severity parameters. Following WLL and granulocyte-macrophage colony-stimulating factor (GM-CSF) inhalation, serum CYFRA21-1 level was significantly reduced, and immunohistochemistry showed CYFRA 21-1 was localized in hyperplastic alveolar type II cells and lipoprotein substances in alveoli. Thus, the serum levels of tumour markers such as CEA, NSE, SCC and CYFRA 21-1 may reflect the severity of disease and predict the therapeutic effect of WLL.

## NSE and acute lung injury

Recently, Gong et al. [127] found that both the expression and activity of PFKFB3, a key glycolytic activator, were markedly increased in lung endothelial cells (ECs) of mice challenged with lipopolysaccharide (LPS) *in vivo* and in LPS-treated human pulmonary arterial ECs (HPAECs) *in vitro*. And blockage of glycolysis by targetting PFKFB3 alleviates sepsis-related acute lung injury (ALI) via suppressing inflammation and apoptosis of ECs. Consistently, the research of Zhong et al. [128] also demonstrated inhibition of glycolysis alleviates LPS-induced ALI in a mouse model in 2016. Similarly, we believe NSE, as an indispensable enzyme in glycolytic pathway, may play a key pathogenic role in ALI. Our research group verified this idea in SD rat model of ALI induced by severe acute pancreatitis (SAP-ALI).

Severe acute pancreatitis (SAP) is an acute abdominal disease with many complications and high mortality [129]. SAP is often complicated with systemic inflammatory response syndrome (SIRS) and multiple organ dysfunction syndrome (MODS), with mortality as high as 10–30% [130]. ALI is one of the most common early complications of SAP and is also the main reason for high mortality in the early stage. Sixty percent of SAP patients die of respiratory failure within 7 days after admission in hospital [131,132]. In recent years, scholars have defined this lung damage as SAP-ALI. Although many researches have focussed on the pathogenesis and drug intervention of SAP-ALI, the exact pathogenesis is still not fully clear, and the mortality rate is still high. We found that in SAP-ALI rat model, the expressions of NSE, caspase-1, IL-1β and TNF-α in injured lung tissue were significantly increased, and NSE inhibitor could significantly inhibit the expression and activation of caspase-1 and alleviate lung injury. This may be related to the abnormal enhancement of glycolysis mediated by NSE (a key isoenzyme of glycolysis) and the promotion of cell pyroptosis mediated by caspase-1 during SAP-ALI [133].

## Conclusion

NSE is considered a multifunctional protein and different cellular localization and interactions with other molecules strongly suggest its multiple cellular engagements: prompting that its traditional name as ‘neuron specific’ may need to be revisited. Increasing empirical evidence show that NSE can play important role in the diagnosis, treatment monitoring and prognosis evaluation of various lung diseases. Advanced understanding of the structure, function, biochemical and clinical characteristics of NSE may provide new ideas for the treatment of these diseases.

## Abbreviations

ACE: antigene carcino embryonnaire
ALI: acute lung injury
ALP: alkaline phosphatase
BALF: bronchoalveolar lavage fluid
CA125: cancer antigen 125
CEA: carcinoembryonic antigen
CGA: chromogranin A
CI: confidence interval
CR: complete relief
CRP: C-reactive protein
CV: coefficient of variation
EBC: exhaled breath condensate
ECM: extracellular matrix
ECOG: Eastern Cooperative Oncology Group
ED: extensive-stage disease
EGFR-TKI: epidermal growth factor tyrosine kinase inhibitor
EIA: enzyme immunoassay
ELISA: enzyme-linked immunosorbent assay
Eno1: α-enolase
FITC: fluorescein isothiocyanate
INF-γ: interferon-gamma
LD: limited-stage disease
LDH: lactate dehydrogenase
LOD: limit of detection
LPS: lipopolysaccharide
mAb: monoclonal antibody
MBP-1: c-myc promoter-binding protein-1
MCP-1: monocyte chemoattractant protein-1
MS: median survival
NNE: non-neuronal enolase
NO: nitrogenmonoxide
NSCLC: non-small cell lung cancer
NSE: neuron-specific enolase
OS: overall survival
PAP: pulmonary alveolar proteinosis
PFKFB3: 6-phosphofructo-2-kinase/fructose-2,6-biphosphatase 3
PI3K: phosphatidylinositol-4,5-bisphosphate 3-kinase
PR: partial relief
ProGRP: pro-gastrin-releasing peptide
RECIST: Response Evaluation Criteria in Solid Tumors
RIA: radioimmunoassay
ROC: receiver operating characterristic
ROS: reactive oxygen species
SAP-ALI: ALI induced by severe acute pancreatitis
SCLC: small cell lung cancer
SD: stable disease
SPN: solitary pulmonary nodule
TB: tuberculosis
TNM: tumor node metastasis
WLL: whole lung lavage
